# Immunohistochemical Expression of Hypoxia-Inducible Factor-1 Alpha in Oral Squamous Cell Carcinoma

**DOI:** 10.7759/cureus.84597

**Published:** 2025-05-22

**Authors:** Narmy Thangjam, Evarisalin Marbaniang, Biswajit Dey, Caleb Harris, Donboklang Lynser, Pranjal Kalita

**Affiliations:** 1 Pathology, North Eastern Indira Gandhi Regional Institute of Health & Medical Sciences (NEIGRIHMS), Shillong, IND; 2 Surgical Oncology, North Eastern Indira Gandhi Regional Institute of Health & Medical Sciences (NEIGRIHMS), Shillong, IND; 3 Radiology, North Eastern Indira Gandhi Regional Institute of Health & Medical Sciences (NEIGRIHMS), Shillong, IND

**Keywords:** depth of invasion, hypoxia-inducible factor-1 alpha, immunohistochemistry, oral cavity squamous cell carcinoma, stage

## Abstract

Oral squamous cell carcinoma (OSCC), a malignant neoplasm of the oral cavity, is commonly observed in the northeastern region of India, likely due to dietary habits, the prevalence of infections, and possibly genetic predisposition. Hypoxia plays a crucial role in regulating tumor proliferation and survival. In the absence of sufficient oxygen, several mechanisms and factors become instrumental in driving tumorigenesis. One such factor is hypoxia-inducible factor-1 alpha (HIF-1α), a key transcription factor from the inducible factor family that regulates gene expression in response to reduced cellular oxygen levels. Despite its significance, studies investigating HIF-1α overexpression in OSCC remain limited, and only a few have explored its correlation with clinical and pathological parameters. This study involved a retrospective analysis of histopathologically confirmed OSCC cases from resected oral specimens collected over a four-year period (2020-2024). HIF-1α overexpression was evaluated in relation to clinical variables such as age and sex and pathological features including histological grade, stage, depth of invasion (DOI), tumor size, lymphovascular invasion, perineural invasion, and worst pattern of invasion. A statistically significant association was observed between HIF-1α overexpression and both higher tumor stage and greater DOI, supporting its link to more aggressive disease behavior. Given the complexity of OSCC, identifying markers that may serve as therapeutic targets is of critical importance. HIF-1α emerges as one such marker - its presence not only indicates a more aggressive tumor phenotype but also suggests potential for future targeted therapies.

## Introduction

Oral cancer ranks as the 16th most frequently occurring cancer worldwide and presents with a broad range of anatomical distribution [1]. The most common histological type affecting the oral cavity is oral squamous cell carcinoma (OSCC), which accounts for 2-4% of all cancer cases globally [2]. India bears the highest burden of oral cancer, contributing approximately 20% to the global incidence [3]. OSCC is more prevalent among males, with the highest incidence observed in individuals aged 40-69 years.

The northeastern region of India reports the highest incidence of oral malignancies, accounting for 54.48% of the national burden. This elevated prevalence is largely attributed to regional dietary habits, the presence of various infectious agents, and unique genetic traits that distinguish the local population from the rest of India [4]. Risk factors such as betel quid and tobacco chewing, along with the consumption of fermented and smoked foods, are strongly associated with the development of OSCC in this region. Genetically, populations in Northeast India show closer affinities with East Asian groups, unlike other regions of the country.

Hypoxia-inducible factor-1 alpha (HIF-1α) is a key transcription factor that regulates gene expression under hypoxic conditions. Through its angiogenic properties, it enables malignant cells to survive and proliferate in low-oxygen environments [5]. In OSCC, elevated HIF-1α expression has been associated with poorer prognosis [6].

This study aimed to evaluate the expression patterns of HIF-1α and its correlation with various clinicopathological parameters. To date, such an investigation has not been conducted in patients with OSCC from the northeastern region of India, which is characterized by its ethnically diverse population.

## Materials and methods

This retrospective analytical study was conducted at a single center over a period of four years, from January 2020 to January 2024. Ethical approval was obtained from the Research and Ethics Committee of North Eastern Indira Gandhi Regional Institute of Health & Medical Sciences (NEIGRIHMS) (NEIGR/IEC/M12/T16/2023). Given the retrospective nature of the study, the requirement for informed consent was waived by the committee.

All H&E-stained slides of oral resected specimens, specifically mandibulectomy, glossectomy, and maxillectomy cases diagnosed as OSCC, were independently reviewed by two histopathologists. Relevant clinical and radiological data were retrieved from the hospital’s medical records department. The histopathologists were blinded to the clinical and radiological information to eliminate bias. Patients who had received chemotherapy or radiotherapy were excluded from the study.

The resected specimens were fixed in 10% neutral-buffered formalin and processed following the institute’s standard protocol. Automated tissue processing was performed, and H&E-stained slides were used for diagnosis. Reporting followed the College of American Pathologists protocol. Tumors were histologically graded as well-differentiated, moderately differentiated, or poorly differentiated. Tumor staging was performed according to the American Joint Committee on Cancer (AJCC) guidelines. Parameters such as depth of invasion (DOI), worst pattern of invasion (WPOI), lymphovascular invasion (LVI), and perineural invasion (PNI) were assessed as per standard guidelines.

Formalin-fixed, paraffin-embedded tissue sections were cut at 4 µm thickness and incubated at 56°C overnight. Deparaffinization was carried out with three xylene washes (six minutes each), followed by rehydration through graded alcohols (95%, 80%, and 70%), each for six minutes. Slides were then rinsed under tap water, and endogenous peroxidase activity was blocked using 0.3% hydrogen peroxide in methanol for 15 minutes, followed by additional washing.

Antigen retrieval was performed using a microwave (10 minutes at 450 W), after which the slides were allowed to cool to room temperature (approximately 20 minutes). A power block was applied for 10 minutes. Primary antibody HIF-1α (BioGenex EP 118, BioGenex Laboratories, Fremont, California, USA) was incubated for one hour, followed by three washes with Tris-buffered solution (TBS). A super-enhancer was applied for 20 minutes, followed by three additional TBS washes. The HRP reagent was added for 30 minutes, followed by washing steps. Diaminobenzidine chromogen was used for 10 minutes to visualize the reaction, followed by TBS and distilled water rinses. Counterstaining was done using Mayer’s hematoxylin for two minutes and rinsed under tap water. The slides were then dehydrated in graded alcohols, cleared in xylene, mounted with dibutylphthalate polystyrene xylene, and labeled.

**Figure 1:**
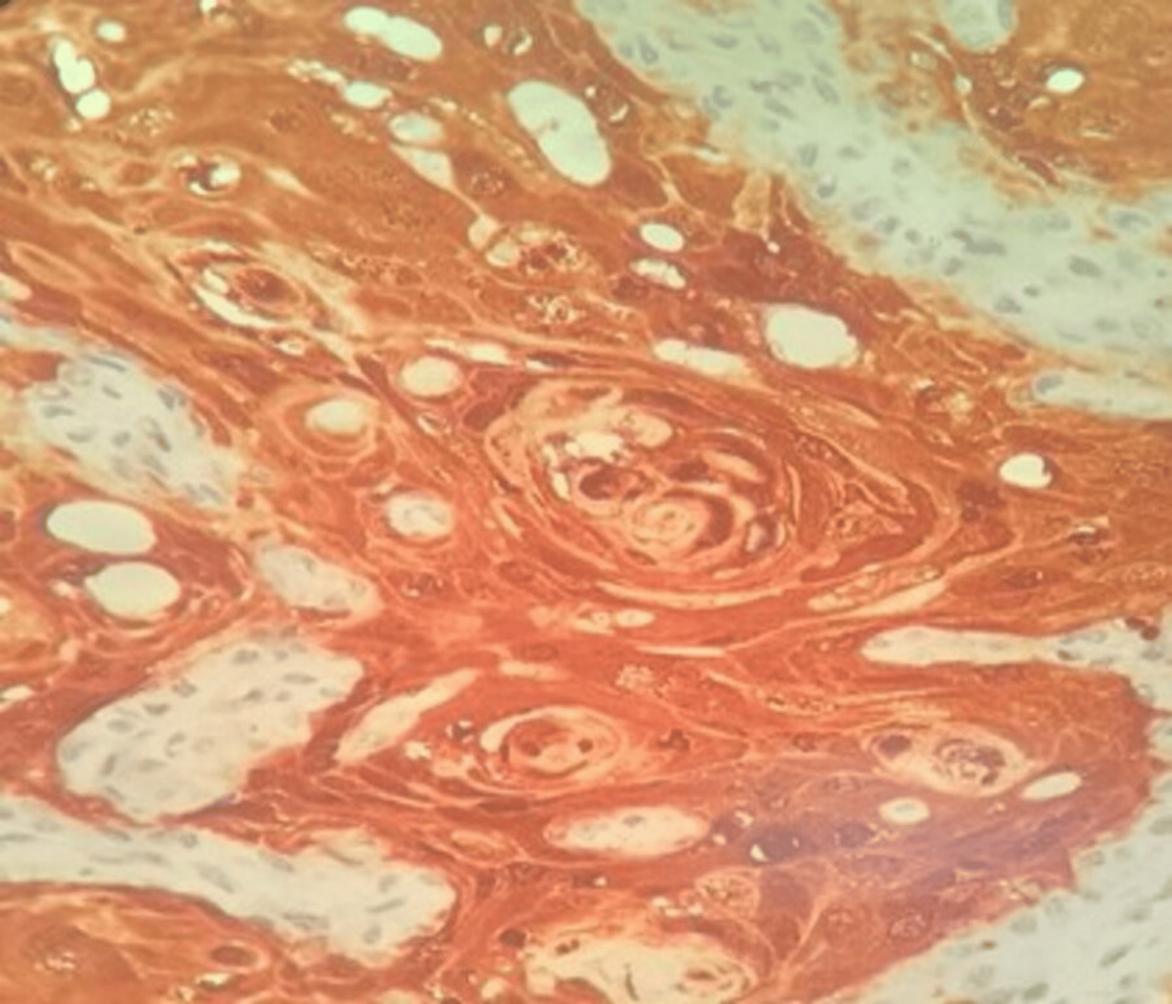
High-power histomorphological image showing squamous cell carcinoma with HIF-1α overexpression (immunoreactive score: 12) (percentage of tumor cells – score: 4; intensity of staining – score: 3) (HIF-1α, IHC, 400× magnification) The immunoreactive score was calculated by multiplying the percentage score of tumor cells by the intensity score. HIF-1α, hypoxia-inducible factor-1 alpha; IHC, immunohistochemistry

**Figure 2:**
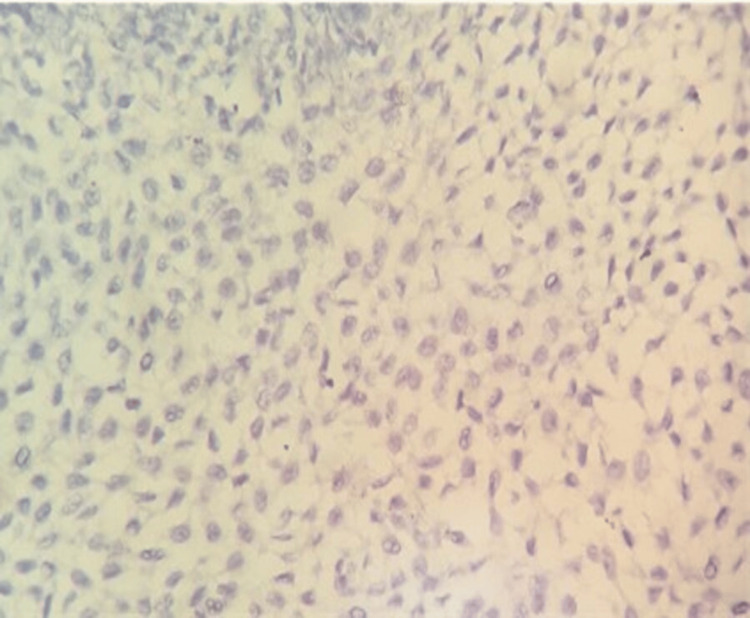
High-power histomorphological image of squamous cell carcinoma showing HIF-1α underexpression (immunoreactive score: 1) (percentage of tumor cells – score: 1; intensity of staining – score: 1) (HIF-1α, IHC, 400× magnification) The immunoreactive score was calculated by multiplying the percentage score of tumor cells by the intensity score. HIF-1α, hypoxia-inducible factor-1 alpha; IHC, immunohistochemistry

A minimum of 100 neoplastic cells was evaluated per case. Immunostaining results were assessed based on both nuclear and cytoplasmic staining patterns, along with staining intensity. An immunoreactive score was calculated by multiplying the score for the percentage of tumor cells by the score for staining intensity (Table 1, Figure 1, Figure 2).

**Table 1:** Percentage, intensity, and immunoreactive score of HIF-1α expression A score of <4 was considered indicative of underexpression, while a score of ≥4 was considered indicative of overexpression of HIF-1α. HIF-1α, hypoxia-inducible factor-1 alpha

Parameters for immunoreactive score	Score
Percentage of tumor cells (%)	
1-10	1
11-50	2
51-80	3
>80	4
Intensity	
Negative	1
Moderate	2
Intense	3
Immunoreactive score (percentage of tumor cells score x intensity score)	
Underexpression	<4
Overexpression	≥4

A score of less than 4 was considered indicative of underexpression, while a score of 4 or greater was considered overexpression [7]. Normal breast tissue served as the negative control, and invasive breast carcinoma cells were used as the positive control in the HIF-1α immunohistochemistry.

Categorical or nominal variables were presented as percentages and proportions. The chi-square test with Yates's correction was applied to evaluate the independence between two categorical variables. Statistical analysis was performed on bivariate variables, with a p-value below 0.05 considered statistically significant. All analyses were conducted using the Social Science Statistics online calculator.

## Results

This retrospective study was conducted over four years, from January 2020 to January 2024, in the Department of Pathology at the NEIGRIHMS in Shillong, India. It included 45 histologically confirmed cases of OSCC.

Among these 45 cases, 25 were males (55.6%) and 20 were females (44.4%), resulting in a male-to-female ratio of 1.25:1. The youngest patient was 33 years old, and the oldest was 78 years old, with a median age of 55 years.

The most common site of OSCC was the buccal mucosa, accounting for 13 cases (29%) (Table 2). Regarding tumor differentiation, 26 cases (57.8%) were well-differentiated squamous cell carcinoma, and 19 cases (42.2%) were moderately differentiated. No poorly differentiated cases were observed.

**Table 2:** Tumor categorization based on site Data are presented as numbers (n) and percentages (%).

Tumor site	Number of cases (n = 45)
Buccal mucosa	13 (28.9%)
Lateral border of the tongue	9 (20%)
Gingivobuccal sulcus	6 (13.3%)
Alveolus	5 (11.1%)
Gingiva	4 (8.9%)
Hard palate	3 (6.7%)
Floor of the mouth	3 (6.7%)
Retromolar trigone	2 (4.4%)
Total	45

For tumor staging, T4 was the most frequent T-stage, observed in 25 cases (55.6%), followed by T2 in 11 cases (24.4%), T1 in five cases (11.1%), and T3 in four cases (8.9%). In nodal staging, 25 cases (55.6%) were classified as N0, 11 cases (24.4%) as N2, five cases (11.1%) as N1, and four cases (8.9%) as N3. No distant metastases were detected in any case. Tumors were staged according to the AJCC staging protocol, with five cases (11.1%) in stage I, 11 cases (24.4%) in stage II, four cases (8.9%) in stage III, and 25 cases (55.6%) in stage IV.

In this cohort of 45 cases, 28 (62.2%) were from the indigenous Khasi population. The Naga community accounted for six cases (13.3%), while Assamese and Nepali patients each contributed four cases (8.8%). Garo, Mizo, and Bihari individuals each represented one case (2.2%).

**Figure 3:**
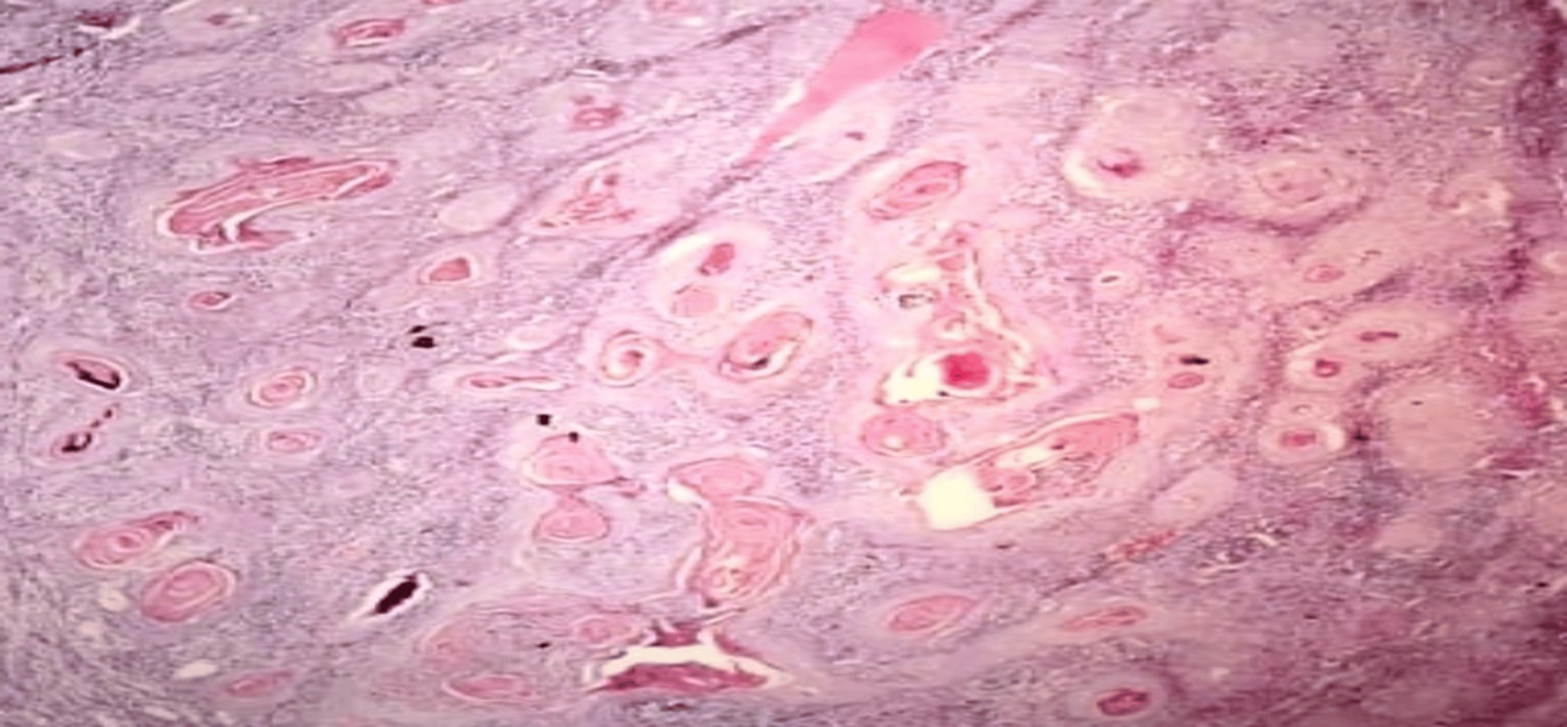
Low-power histomorphology showing invasive squamous cell carcinoma (H&E, 100×)

**Figure 4:**
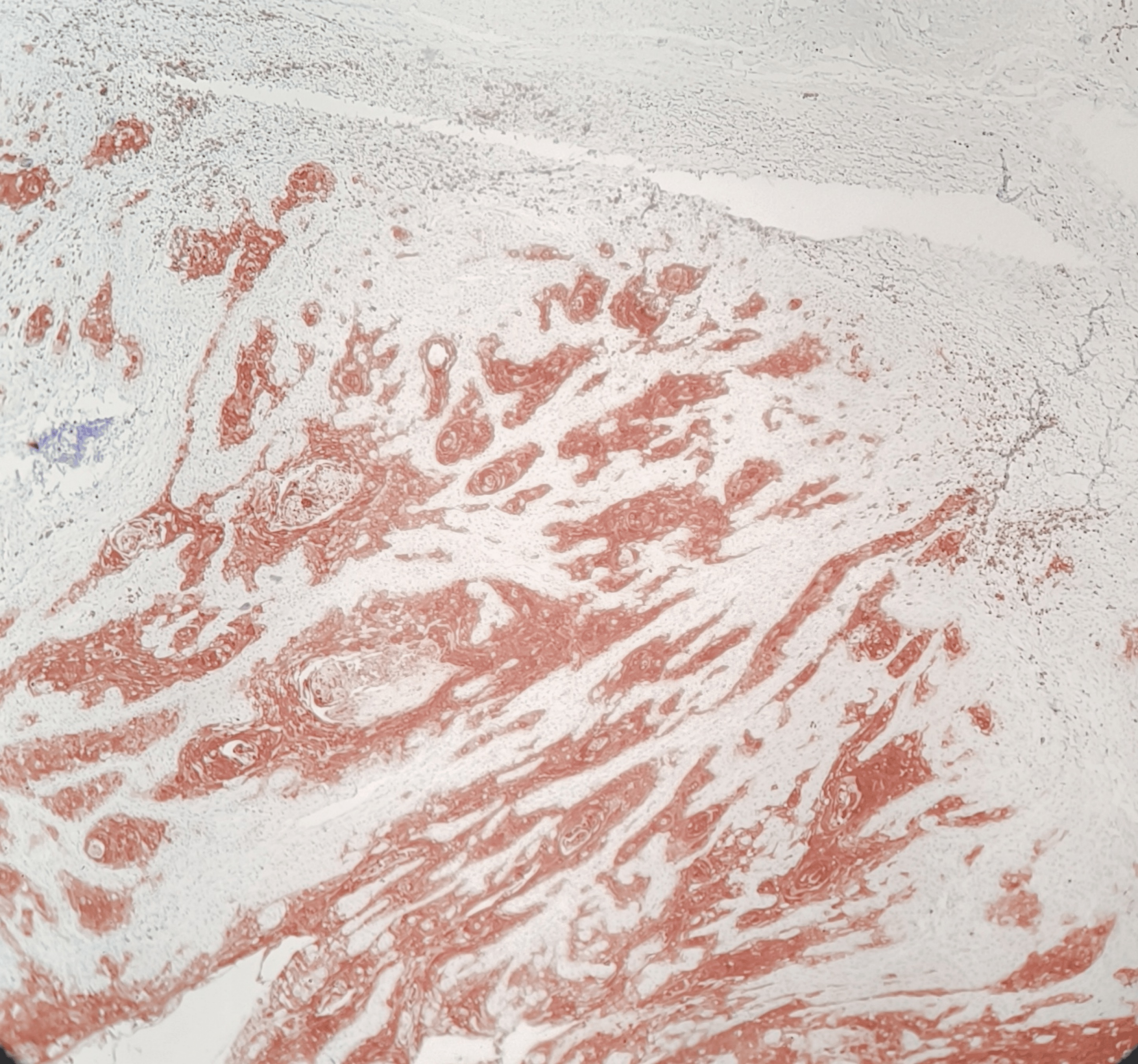
Low-power histomorphology showing HIF-1α overexpression in invasive squamous cell carcinoma (HIF-1α, IHC, 100×) HIF-1α, hypoxia-inducible factor-1 alpha; IHC, immunohistochemistry

HIF-1α overexpression was observed in 30 cases (67%), with the highest frequency among Khasi patients - 18 of 28 cases (64%). Among the HIF-1α-positive Khasi patients, 13 cases (72%) showed higher tumor stage and a DOI greater than 5 mm. The histomorphology and immunohistochemistry findings of an invasive OSCC case are shown in Figure 3 and Figure 4.

A DOI greater than 5 mm was observed in 32 cases (71.1%), while a DOI less than 5 mm was noted in 13 cases (28.9%). LVI was absent in 23 cases (51.1%) and present in 22 cases (48.9%). Similarly, PNI was absent in 32 cases (71.1%) and detected in 13 cases (28.9%). Cases were classified into five WPOIs, ranging from WPOI-1 to WPOI-5, with their distribution detailed in Table 3.

**Table 3:** Categorization of cases based on the WPOI Data are presented as numbers (n) and percentages (%). WPOI, worst pattern of invasion

WPOI	Number of cases (n = 45)
WPOI-1	1 (2.2%)
WPOI-2	6 (13.3%)
WPOI-3	9 (20%)
WPOI-4	16 (35.6%)
WPOI-5	13 (28.9%)

Out of 45 cases, 30 (66.7%) showed HIF-1α overexpression, while 15 cases (33.3%) exhibited underexpression. The relationship between various clinicopathological parameters and HIF-1α immunostaining is summarized in Table 4. Among these parameters, HIF-1α expression was significantly associated with tumor stage (p = 0.036) and DOI (p = 0.027).

**Table 4:** Correlation of HIF-1α immunostaining with clinicopathological parameters The p-value was calculated using the chi-square test with Yates’s correction. A p-value less than 0.05 was considered statistically significant. DOI, depth of invasion; HIF-1α, hypoxia-inducible factor-1 alpha; LVI, lymphovascular invasion; PNI, perineural invasion; WPOI, worst pattern of invasion

Clinicopathological analysis	HIF-1α overexpression	HIF-1α underexpression	Chi-square statistic with Yates’s correction	p-value
Age				
<60 years	19	11	0.113	0.737
>60 years	11	4		
Sex				
Male	19	6	1.361	0.243
Female	11	9		
Histological grade				
Well-differentiated	17	9	0.011	0.915
Moderately differentiated	13	6		
Staging				
Stage I and Stage II	7	9	4.376	0.036
Stage III and Stage IV	23	6		
DOI				
<5 mm	5	8	4.881	0.027
>5 mm	25	7		
Tumor size				
T1 and T2	9	3	0.128	0.721
T3 and T4	21	12		
LVI				
Identified	14	8	0.011	0.916
Not identified	16	7		
PNI				
Identified	9	4	0.013	0.907
Not identified	21	11		
WPOI				
1-3	10	6	0.012	0.912
4-5	20	9		

## Discussion

Semenza et al. first described hypoxia-inducible factor in 1992 and were later awarded the 2019 Nobel Prize in Physiology or Medicine for discovering this critical transcription factor that regulates gene expression in response to decreased cellular oxygenation [8]. HIF-1α, HIF-2α, and HIF-3α are the three oxygen-sensitive subunits, while HIF-1β is oxygen-insensitive. Although HIF-1α and HIF-2α share similar domain architectures and undergo comparable proteolytic regulation, HIF-2α expression is more tissue-restricted. The function of HIF-3α remains unclear, and it is less closely related to the other isoforms [9].

Angiogenesis plays a vital role in both physiological and pathological processes, including cancer. It is a complex, multistep, and temporally regulated process involving numerous genes, modifiers, and pathways. HIF-1α directly activates many genes, such as those involved in matrix metabolism (e.g., urokinase-type plasminogen activator receptor), nitric oxide synthases, and angiogenic and vascular growth factors like vascular endothelial growth factor. While some of these genes may also be regulated independently by hypoxia and secondary mechanisms, HIF-1α is essential for proper vascularization during mouse embryogenesis [10].

Key features of cancer metabolism - such as increased glucose uptake, reduced respiration, and lactate production - are heavily influenced by HIF-1α. HIF activation is common in malignancies and tends to be more pronounced in aggressive tumors, serving as an independent marker of poor prognosis in several cancer types [11].

OSCC ranks eighth globally in cancer incidence, accounting for approximately 4.5% of all cancer cases, according to the 2020 GLOBOCAN estimates. It causes over 450,000 deaths annually, representing about 4.6% of all cancer-related fatalities worldwide. OSCC primarily affects individuals over 50 years old, with a higher prevalence in males. In many countries, the incidence is rising, especially among younger populations. By 2030, it is projected to increase by 30% annually, partly due to lifestyle changes like increased tobacco and alcohol use in developing nations [12].

Tang et al. studied the role of HIF-1α in esophageal carcinoma and found that HIF-1α likely promotes cancer cell proliferation by activating the gene TCF4/TCF7L2, which in turn triggers the Wnt/β-catenin pathway [13].

Our study investigated the role of HIF-1α in OSCC and its correlation with clinicopathological parameters in the northeastern Indian population - the first such study in this region. Most patients were in their fifth or sixth decade of life, with males outnumbering females, confirming male predominance and older age as major risk groups. Among the 45 OSCC cases, 25 (55.6%) were Stage IV, 32 (71.1%) had a DOI greater than 5 mm, and 29 (64.4%) were classified as WPOI-4 or WPOI-5, indicating advanced disease at presentation. HIF-1α expression was assessed in tumor sections from all histopathologically confirmed OSCC cases, revealing overexpression in 30 cases (66.7%) and underexpression in 15 cases (33.3%).

The northeastern region of India is ethnically diverse. A high incidence of OSCC was noted among the Khasi population, accounting for 62% of cases, likely due to dietary habits and the study’s location in a Khasi-dominated area. HIF-1α overexpression was observed in 18 Khasi patients, 13 of whom had higher tumor grade and DOI >5 mm, supporting the association between HIF-1α overexpression and advanced disease at diagnosis.

In our study, HIF-1α overexpression significantly correlated with advanced tumor staging and greater DOI. Zhou et al. reported a strong association between HIF-1α overexpression and advanced TNM stage, consistent with our findings [14]. Similarly, Uehara et al. found higher HIF-1α levels, especially in stage III and IV tumors [15]. Notably, our study revealed a significant link between HIF-1α overexpression and DOI, a correlation not previously emphasized in the literature. No significant associations were found between HIF-1α expression and other clinicopathological parameters such as tumor size (highlighted by Sumera et al.), age, sex, histological grade, LVI, or PNI [6]. The variability in findings may stem from factors including sample size, tumor heterogeneity, differences in immunohistochemistry protocols, alternative hypoxia pathways, and the tumor microenvironment.

Nonetheless, the consistent association of HIF-1α overexpression with higher stage and deeper invasion across multiple studies suggests it is linked to aggressive tumor behavior. Overexpression of HIF-1 is also known to correlate with poorer outcomes and reduced responsiveness to chemotherapy and radiotherapy in various cancers. In the era of targeted therapy, Bui et al. described HIF-1 as a promising target for novel cancer treatments, drug resistance management, and alleviation of cancer-related pain [16]. Therefore, we believe that further research into HIF-1 and the development of specific targeted therapies could have important prognostic and therapeutic implications for OSCC patients presenting with advanced disease.

Limitations

The study’s small sample size and single-center, retrospective design limit the generalizability of the results. The absence of follow-up or survival data restricts the evaluation of HIF-1α as a prognostic marker. Potential inter-observer variability in immunohistochemistry scoring and technical differences in staining protocols may have influenced the findings. Additionally, focusing solely on HIF-1α without assessing other hypoxia-related markers limits a comprehensive understanding of tumor hypoxia in OSCC.

## Conclusions

OSCC is a complex tumor influenced by multiple factors. HIF-1α is a key regulator of the tumor hypoxic environment, promoting tumor survival, and may serve as a potential therapeutic target. In our study, HIF-1α overexpression was associated with higher tumor stage and increased DOI, indicating a more aggressive disease at presentation.
